# Eco-friendly management of cercospora leaf spot disease in sugar beet using ZnO and MgO nanoparticles

**DOI:** 10.1186/s12870-026-09965-x

**Published:** 2026-10-12

**Authors:** Heba‑Alla S. AbdElfatah, Nashwa M. A. Sallam, Hadeel M. M. Khalil Bagy, Mohamed S. Mohamed, Mennatallah M. A. El‑Geddawy, Rofida F. Moftah, Mohamed Abd El-Aal

**Affiliations:** 1 Department of Plant Pathology, Faculty of Agriculture, Assiut University, Assiut, Egypt; 2 Department of Food Science and Technology, Faculty of Agriculture, Assiut University, Assiut, Egypt; 3 Department of Chemistry, Faculty of Science, Assiut University, Assiut, Egypt

**Keywords:** Sugar beet, Cercospora Leaf Spot (CLS), ZnO, MgO, NPs, Root yield, Sucrose content

## Abstract

**Background:**

*Cercospora* leaf spot, caused by *Cercospora beticola*, is the most destructive disease of sugar beet. This study evaluated zinc oxide (ZnO) and magnesium oxide (MgO) nanoparticles (NPs) as sustainable alternatives to chemical fungicides.

**Results:**

In vitro experiments, at a concentration of 90 ppm, the fungal inhibition rate of MgO NPs reached 58.33%, higher than the 52.08% recorded for ZnO NPs. SEM observations confirmed that both types of nanoparticles caused severe distortion and deformation of the mycelium of the tested fungi. However, under greenhouse and field conditions, ZnO NPs provided superior disease control, reducing disease severity by up to 50.92%. Both also raised crop phenolic content and boosted PO, PPO, and PAL enzyme activity, peaking 6 days post-treatment. Treated crops showed higher root yield and sucrose and less unwanted sugar than untreated controls.

**Conclusion:**

Both ZnO and MgO nanoparticles represent promising alternatives to conventional fungicides for the management sugar beet leaf spot disease. Although MgO NPs were more effective in vitro, ZnO NPs were more effective in greenhouse and field conditions. They can also enhance sugar beets’ defense responses, root yield, and sucrose content, and can be incorporated into the sustainable plan for integrated sugar beet disease management.

## Introduction

Sugar beet (*Beta vulgaris L.*) has become an important crop in Egypt as the country seeks to achieve self-sufficiency in sugar production and increase sugar exports. According to the latest statistics in reference [1], Egypt has currently developed a certain scale of sugar beet planting area and annual output. The pathogen causing Cercospora leaf spot (CLS) in sugar beets is *C. beticola*, which can reduce sucrose content by as much as 50% [2]. The mainstream traditional prevention and control strategies for CLS rely on synthetic fungicides. Multiple previous studies have confirmed that this approach can effectively suppress the disease, but it also has three unavoidable core flaws. First, the economic cost of continuous input keeps rising. Second, pesticide residues easily cause severe environmental pollution. Third, long-term application has forced the pathogen to gradually develop resistance. The current status has prompted the academic community to seek sustainable alternative control pathways [2]. Nanoparticle technology, featuring high disease control efficiency and low environmental impact, has become an emerging feasible solution in the field of plant protection [3, 4]. Among the relevant materials, the two metal oxide nanomaterials that this study focuses on zinc oxide nanoparticles (ZnO NPs) and magnesium oxide nanoparticles (MgO NPs), have been verified in multiple independent studies to exhibit strong antimicrobial properties, chemical stability under harsh environmental conditions, and safety for application [5, 6]. They can also activate three core defense enzymes in plants: phenylalanine ammonia-lyase (PAL), peroxidase (PO), and polyphenol oxidase (PPO) [7, 8]. By promoting the synthesis of phenolic compounds, reinforcing cell walls, and inducing the production of antimicrobial substances, they reduce disease severity and improve plants’ overall defense capacity [9]. The use of nanotechnology in the sustainable treatment of plant diseases is currently receiving increasing global interest. However, research on the use of metal oxide nanoparticles to control sugar beet Cercospora leaf spot remains extremely limited. While existing studies have demonstrated that zinc oxide (ZnO) nanoparticles exhibit antifungal activity against a wide range of plant-pathogenic fungi, no research has directly compared the efficacy of ZnO and MgO NPs as controls against *C. beticola*, that causes this disease. No prior work has simultaneously evaluated the application performance of these two groups of nanomaterials in both greenhouse and field settings. The two types of nanoparticles differ in their physicochemical properties, antifungal mechanisms, and interactions with plant tissues and these differences directly affect their disease control outcomes and crop-production performance. This study is the first effort to comprehensively compare the two types of nanoparticles for controlling this disease, and it simultaneously assesses their antifungal activity, disease severity, plant growth, crop yield, and crop biochemical responses to provide new approaches for the sustainable management of sugar beet diseases.

Therefore, this study sets the following objectives: synthesize the two types of nanoparticles, evaluate their in vitro inhibitory effect on the growth of *C. beticola*at different concentrations, analyze their antimicrobial mechanism using Scanning Electron Microscope (SEM), assess their impact on CLS severity under greenhouse conditions, test changes in phenolic content and the activity of the three defense enzymes in plant tissues, and finally evaluate the field control efficacy of the two nanoparticles, including their effects on crop yield and the sugar content of storage roots.

## Materials and methods

### Source of the pathogenic isolate

The pathogenic isolate of *C. beticola* utilized in this study, which causes CLS in sugar beet, was originally sourced from the Department of Plant Pathology, Faculty of Agriculture, Assiut University, Egypt. This specific isolate was previously characterized and submitted to GenBank under the accession number PQ900242.1, as described by [10].

### Synthesis of ZnO and MgO Nanoparticles (NPs)

#### Materials

ZnO and MgO NPs were synthesized using analytical grade chemicals. All solutions were prepared using bi-distilled water. The precursors used were anhydrous zinc chloride (ZnCl_2_; Assay: 98%, Advent Chembio Pvt ltd.), magnesium chloride hexahydrate (MgCl_2_.6H_2_O; Assay: 98%, Alpha Chemika), and sodium hydroxide (NaOH, Research-Lab Fine Chem Industries).

#### Synthesis procedures of NPs

ZnO NPs were synthesized by dissolving 33.5 g of ZnCl_2_ in 500 mL of distilled water, followed by heating at 80 °C under continuous stirring [11, 12]. For MgO NPs, as described by [13], 100.9 g of MgCl_2_·6 H₂O was dissolved in the same volume of distilled water and treated under identical conditions. In both cases, an NaOH solution (8 g in 100 mL of distilled water) was added dropwise to the metal chloride solutions until the pH reached 10, resulting in a white suspension. The reaction mixtures were maintained at 80 °C for 3 h and then allowed to cool to room temperature for 2 h. The resulting precipitates were collected by filtration, washed thoroughly with distilled water and ethanol, dried overnight at 80 °C, and finally calcined at 500 °C for 3 h.

### NPs characterization

The structure and crystallinity of the nanoparticles (MgO and ZnO NPs) were analyzed using X-ray diffraction (XRD) with a Philips diffractometer (PW 2103, Cu Kα, λ = 1.5418 Å). The analysis was conducted over a range of 4° to 80°. Additionally, Fourier transform infrared spectroscopy (FTIR) was performed using a Nicolet spectrophotometer (model 6700) over the range 4000 –400 cm⁻¹ to determine the chemical structures and functional groups of the nanoparticles. Furthermore, the size and shape of the nanoparticles were evaluated using transmission electron microscopy (TEM) with a JEOL Model JSM-5400LV (JEOL, Tokyo, Japan).

### Effect of NPs on the growth of *C. beticola in vitro*

The pathogenic isolate (*C. beticola*) was cultured on PDA medium 7 days prior to subsequent experiments. Aqueous suspensions of ZnO and MgO NPs were prepared at 10, 30, 60, and 90 ppm, corresponding to 0.01, 0.03, 0.06, and 0.09 g/L, respectively. A mycelial disc (5 mm) from a 7-day-old culture of the pathogen was placed at the center of PDA plates containing different NPs concentrations. Control plates were inoculated with the pathogen alone, without NPs. Each treatment was replicated three times. The plates were incubated at 27 °C ± 1 °C for a week. The growth inhibition percentage of the pathogen was determined by comparison with fungal growth in control plates [14] as follows:

$$\text{Growth inhibition}\left({\%}\right)=\frac{C-h}{C}\times{100}$$

C = Diameter of mycelial growth in the control plate (cm)

h = Diameter of mycelial growth in the plate treated with NPs (cm)

### Scanning Electron Microscopy (SEM) analysis of NPs with *C. beticola*

SEM was used to investigate the structural effects of ZnO and MgO NPs (90 ppm) on C. *beticola* hyphae and spores in vitro. The experiment was conducted at the Electron Microscope Unit (EMU), Assiut University. A 5 mm mycelial disc taken from a 7-day-old culture was inoculated onto PDA plates amended with the respective nanoparticles, while control plates contained only the pathogen. All plates were incubated at 27 ± 1 °C for seven days. After incubation, mycelial samples from both treated and control groups were collected and examined using a JEOL JSM-5400 LV scanning electron microscope operated at 15 kV to observe morphological and ultra-structural alterations in fungal hyphae and spores [15].

### Effect of NPs on CLS disease of sugar beet under greenhouse conditions

#### Preparation of pathogen inoculum

To produce spore suspensions of C. *beticola*, two 5 mm mycelial discs were taken from a 7-day-old culture and inoculated into a 1000 mL Erlenmeyer flask containing sugar beet leaf extract dextrose agar (SBLEDA) medium. The medium consisted of 100 g sugar beet leaf extract, 20 g dextrose, and 15 g agar per liter. Cultures were incubated at 27 ± 2 °C under continuous shaking at 120 rpm for 7 days. The resulting growth was harvested by filtration through filter paper, and the collected biomass was homogenized in sterile distilled water using a Waring blender. Finally, the propagule suspension was adjusted to a concentration of 1 × 10⁷ CFU/mL [10].

#### Preparation of NPs suspension

Each type of ZnO and MgO NPs was applied at the optimal concentration (90 ppm), which showed the highest inhibition of *C. beticola in vitro*. The required amounts of each NPs were suspended in sterile distilled water in Erlenmeyer flasks (1000 mL) and thoroughly mixed to ensure complete homogeneity before use in the greenhouse experiment.

#### Plant material

Sugar beet seeds (*Beta vulgaris* L. cv. Hossam) were obtained from Sugar Crops Research Institute, Agricultural Research Center (ARC), Giza, Egypt. The cultivar identity was verified based on the official records and seed certification provided by the Institute.

### Evaluation of CLS disease severity

This study was carried out in the Plant Pathology Department, Faculty of Agriculture, Assiut University, Assiut, Egypt. Three sterilized seeds were sown directly in each pot (30 cm in diameter) filled with autoclaved sandy clay soil (3 kg/pot), and 3 g of fertilizer NPK 12:4:6 was added to each pot. The pots were kept at 30 ± 5 °C with 75–90% relative humidity and watered whenever needed. Plants were infected with a suspension of *C. beticola* at 60 days of age [10]. Ten days after needed, plants were treated with MgO NPs and ZnO NPs twice, with a 10-day interval between applications. The treatments were applied using a fine-mist hand-held pressure sprayer (30 mL per plant). Control plants received distilled water only. All plants were covered with polyethylene bags for 48 h after each application to maintain high humidity and facilitate infection. Disease severity was assessed 30 days after treatment by using the disease scales [16] as follows: 0 = no leaf lesions, 1 ≥ 25% few scattered spots (5–10 per leaf), minimal yellowing., 2 ≥ 50% moderate infection, many spots (20–50 per leaf), some leaf yellowing, 3 ≥ 75% sever infection, extensive necrosis and leaves wilting., and 4 ≥ 100% majority of the leaf surface covered with necrotic spots, leaves collapsing.

The formula:

$$\begin{aligned}&\text{Disease severity}\left(\%\right)=\\&\Sigma\left(\mathrm{n}\times\mathrm{r}\right)/\left(\mathrm{N}\times\mathrm{R}\right)\times{100}\end{aligned}$$

where: n = number of infected leaves on the plant, r = numerical rating of infected leaves, N = total number of leaves on the plant, and R = maximum numerical rating.

### Biochemical analysis of phenolic compounds and defense-related enzyme activity

This study aimed to assess the effects of NPs on biochemical responses and phenolic compounds in sugar beet plants. Leaf samples used for the determination of total phenolic content and PO, PPO, and PAL were collected from the greenhouse experiment. Fully expanded leaves were sampled from each treatment at the specified times 0 (day of treatment), 3, 6, and 9 days after the application of each type of nanoparticle. Leaves inoculated only with the pathogen served as the diseased control, while non-inoculated leaves served as the healthy control. Each treatment was conducted in triplicate. Then, they were immediately transferred for biochemical analyses.

#### Preparation of samples

For each treatment, fresh leaf samples were immediately immersed in liquid nitrogen and stored at − 80 °C until analysis of total phenolic content and enzyme activities. Each treatment was conducted in three replicates.

#### Determination of total protein content

The total protein content in sugar beet leaves was determined using the Bradford method [17]. Bradford reagent was prepared by dissolving 100 mg of Coomassie Brilliant Blue G-250 in 50 mL of 95% ethanol, then adding 100 mL of 85% H₃PO₄. The mixture was then diluted to 1000 mL with distilled water, filtered, left to stand for 24 h, and stored at 4 °C. For leaf sample extract preparation, (0.5 g of fresh sugar beet leaves were homogenized in 2 mL of phosphate buffer (pH 7.0), centrifuged at 10,000 g for 15 min at 4 °C, and the supernatant was collected. For the assay, 100 µL of the leaf sample supernatant was mixed gently with 1.5 mL of Bradford reagent and incubated for 15 min at room temperature. Absorbance was measured at 595 nm using a spectrophotometer (Spectronic^®^ 20 Genesys™, Schutt Labortechnik).

### Estimation of total phenolic content

Total phenolic compound content was determined using the method described by [18]. Leaf samples (0.5 g) were homogenized in 5 ml 80% methanol and incubated in a water bath at 70 °C for 1 h. The resulting methanolic extract was mixed with distilled water, Folin-Ciocalteu reagent, and sodium carbonate (Na_2_CO_3_) at room temperature, and the mixture was allowed to stand for 20 min. Absorbance was measured at 725 nm, and phenolic content was calculated using a standard gallic acid curve and expressed as mg/g FW.

### Determination of enzyme activity

#### Peroxidase activity (PO)

Leaf samples from each treatment (0.5 g) were homogenized with 0.1 M Na-acetate buffer (10 mL buffer /gram of fresh leaf tissue). The homogenates were centrifuged at 10,000 rpm for 30 min at 4 °C. The resulting supernatants were used for the enzyme assay. Each treatment was analyzed in three replicates. Following the method of [18], the assay was performed spectrophotometrically using guaiacol as a substrate. The reaction mixture contained 0.2 mL of supernatant, 1 mL of 0.1 M sodium acetate buffer (pH = 5.2), 0.2 mL of 1% guaiacol, and 0.2 mL of 1% H₂O₂. The mixture was incubated at 25 °C for 5 min, and the absorbance was measured at 436 nm. The extraction buffer served as the blank. Enzyme activity was calculated based on the change in absorbance and expressed as enzyme units/mg protein.

#### Polyphenol Oxidase (PPO)

The activity of PPO was determined following the method of [18]. Leaf samples (0.5 g) from each treatment were homogenized with 0.1 M Na-acetate buffer (10 mL buffer /gram of fresh leaf tissue). The homogenates were centrifuged at 10,000 rpm for 30 min at 4 °C. The reaction mixture contained 0.5 mL supernatant, 2 mL (50 mM) Sorensen phosphate buffer (pH 6.5; prepared by dissolving 6.8 g KH₂PO₄ and 8.99 g Na_2_HPO₄·2 H₂O in 1000 mL distilled water, with 0.372 g EDTA added), and 0.5 mL benzyl catechol substrate. The mixture was incubated at 37° C for 2 h, and the absorbance was measured at 410 nm. PPO activity was expressed as enzyme units per mg protein.

#### Phenylalanine Ammonia-lyase (PAL)

The activity of phenylalanine ammonia-lyase (PAL) was determined according to [18] with minor modifications. A 0.5 g frozen leaf sample from each treatment was homogenized in 5 mL of potassium phosphate buffer (100 mM, pH 7.5) containing 0.5 mL ethylenediaminetetraacetic acid (EDTA) and 0.5 g polyvinylpyrrolidone (PVP). The homogenate was then centrifuged at 10,000 rpm using a Centrifuge 5415 (Eppendorf) for 30 min at 4 °C. The supernatant was used as an enzyme extract. The extract (0.5 mL) was mixed with (2 mL) borate buffer (80 mM, pH 8.9) and phenylalanine (1.5 mL) and incubated at 30 C° for 1 h, following which HCl (0.5 mL) was added. The trans-cinnamic acid content was measured at 290 nm, and the activity of PAL was expressed as U/mg protein.

### Effect of NPs under field conditions

Field experiments were conducted at the Experimental Farm of the Plant Pathology Department, Faculty of Agriculture, Assiut University, Assiut, Egypt, during growing seasons 2023–2024 and 2024–2025.

Field plots (3 × 3.5 m) included three rows (each 3 m in length, with 3 plants/row). Each row contained 3 seeds and were arranged in a completely randomized block design. 60 days after planting, sugar beet plants (cv. Hossam) were inoculated with a suspension of *C. beticola* as described previously. Ten days after inoculation, the infected plants were treated with the respective NPs suspensions, following the same procedures used in the greenhouse experiments. Healthy control plants received only distilled water. For each treatment, three plots served as replicates. Disease severity was assessed 30 days after the final treatment, as described by [14].

### Assessment of sugar beet root yield

Sugar beet roots were harvested after six months in both growing seasons. Root yield was determined by weighing all harvested roots from each plant in a plot. Data from all plants in each plot were averaged, and yield was expressed in tons per feddan [19].

### Analysis of sugar contents in sugar beet roots

All analyses of sugar content (reducing sugars and sucrose) were conducted at the Food Science and Technology Department, Faculty of Agriculture, Assiut University.

#### Sample preparation

Six months after planting, sugar beet roots from each treatment (healthy plants, diseased plants, and treated plants with NPs) in both growing seasons were collected. Each treatment included three replicates. The roots were carefully washed to remove soil residues. Then, 100 g of each sample was chopped and homogenized with 100 mL of distilled water for 3 min using a mixer. The mixtures were first filtered to obtain sugar beet juice. Subsequently, 5 mL of a 10% lead acetate solution was added to each sample to precipitate proteins, pigments, and other non-sugar components. Samples were then refiltered to remove sediments and stored at − 20 °C until further analysis.

#### Assessment of reducing sugars and sucrose

Reducing sugars (glucose and fructose) were measured using the dinitrosalicylic acid (DNS) method as described by [20]. Equal volumes (3 mL) of DNS reagent and the sample were mixed in test tubes and heated in a boiling water bath for 15 min. After heating, 1 mL of 40% potassium sodium tartrate was added to stabilize the color. The tubes were cooled to room temperature, and absorbance was measured at 575 nm using a spectrophotometer. A standard curve was generated using glucose solutions (0.00–0.1%), following the same procedure.

#### Evaluation of sucrose content

Sucrose content was measured utilizing an automatic saccharimeter on a lead acetate basis according to [21].

### Statistical analysis

Three replicates were used in each experiment. The means and standard errors had been estimated for them. Data analyses were performed using two-way ANOVA in SPSS 19.0. Means were compared using Duncan’s multiple-range test, and statistical significance was assessed at *p* ≤ 0.05 [22].

## Results

### ZnO NPs and MgO NPs characterization

**Fig. 1:**
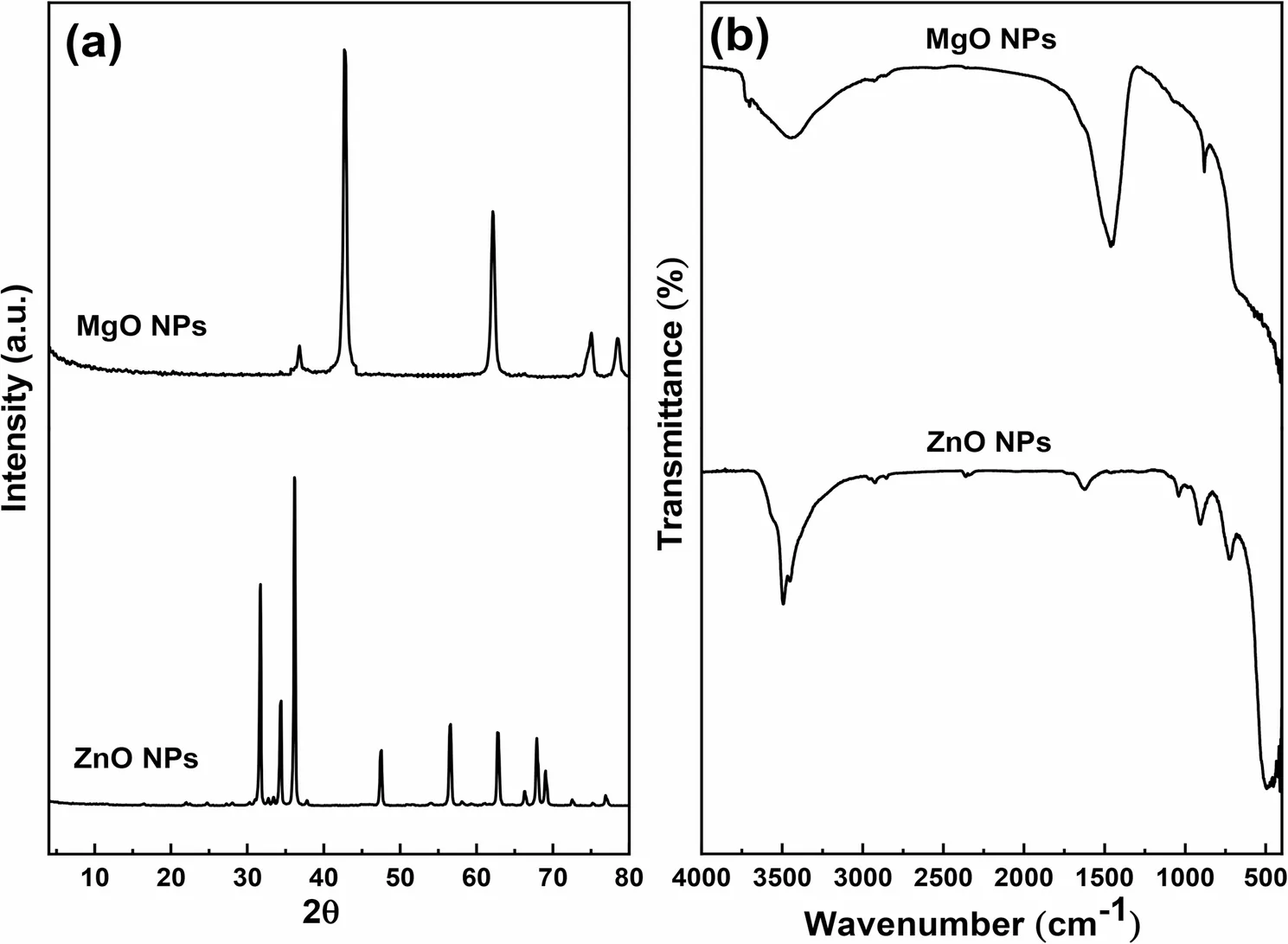
**a** XRD patterns and (**b**) FTIR spectra for ZnO and MgO NPs

The XRD patterns of ZnO NPs and MgO NPs are displayed in Fig. 1a. The pattern of ZnO NPs showed reflections at 2ϴ values of 31.7^o^, 34.4^o^, 36.2^o^, 47.6^o^, 56.6^o^, 62.7^o^, 66.3^o^, 67.9^o^, 69^o^, 72.5^o^, and 76.8^o^ which are attributable to (100), (002), (101), (102), (110), (103), (200), (112), (201), (004), and (202) planes of hexagonal ZnO (JCPDS No. 01-080-0074), respectively. While the pattern of MgO NPs showed diffraction peaks at 2ϴ values of 36.8°, 42.7°, 62.1°, 75°, and 78.5^o^ which correspond to (111), (200), (220), (311), and (222) planes of cubic MgO (JCPDS No. 01-075-0447), respectively. No stray diffraction peaks were detected, confirming the high purity of the samples. Calculations using the Scherrer equation indicate average grain sizes of 33.6 nm and 23.3 nm for the two nanoparticles, respectively.

The FTIR spectra of ZnO NPs and MgO NPs are presented in Fig. 1b. The spectrum for ZnO NPs showed bands at 3493 and 3452 cm^− 1^ (O-H stretching vibration), 2925 and 2852 cm^− 1^ (C-H stretching vibration), 1624 cm^− 1^ (O-H bending mode), 1040 cm^− 1^ (Zn–O–Zn stretching vibration), 905 cm^− 1^, 727.8 cm^− 1^, 494 cm^− 1^, and 452 cm^− 1^ (Zn-O stretching vibrational mode). The spectrum of MgO NPs showed bands at 3701 and 3442 cm^− 1^ (O-H stretching vibration), 1460 cm^− 1^ (O-H bending vibration), 881 cm^− 1^ (Mg-O-Mg bond), and 430 cm^− 1^ (Mg-O bond stretching).

**Fig. 2:**
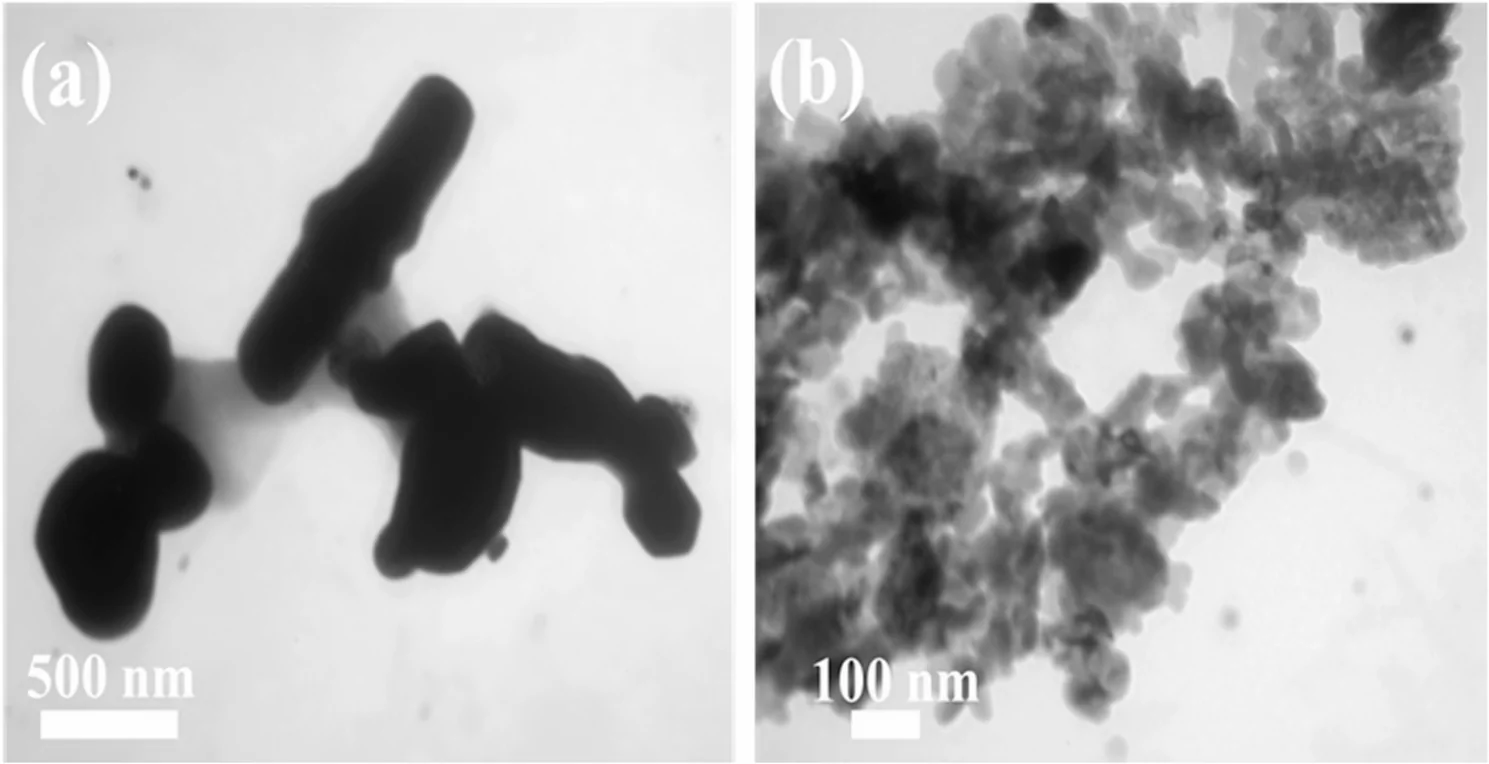
TEM images of (**a**) ZnO and (**b**) MgO NPs

TEM images of ZnO NPs and MgO NPs are presented in (Fig. 2). The micrograph of ZnO NPs showed a mixture of semi-spherical particles with an average diameter of 214 nm and rod-shaped particles with average length and width of 958 and 299 nm, respectively. The image of MgO NPs showed aggregates with an average diameter of 60.5 nm.

### Effect of ZnO and MgO NPs on *C. beticola* growth in vitro

According to the data in Table 1, both ZnO and MgO NPs significantly reduced the mycelial growth of *C. beticola* at all tested concentrations (10, 30, 60, and 90 ppm) compared to the control. The inhibition of the growth of *C. beticola* increased consistently with increasing NPs concentration. The inhibition rates corresponding to each tested NPs concentration were at 90 ppm (52.08% and 58.33%, respectively), followed by 60 ppm (39.52% and 43.75%, respectively). The least impact was noted at 10 ppm (23.33% and 22.5%, respectively). Overall, MgO NPs exhibited a superior inhibitory capacity.

**Table 1:** Effect of Zno NPs and Mgo NPs on *C. beticola* growth in vitro

Treatments		% of growth inhibition		Mean
Nanoparticles	Concentrations (ppm)			
ZnO NPs	10	23.33	G	29.26 b
	30	32.27	F	
	60	39.52	D	
	90	52.08	B	
	Control	0	H	
MgO NPs	10	22.5	g	31.88 a
	30	34.58	E	
	60	43.75	C	
	90	58.33	A	
	Control	0	H	

Means of three replicates in column designated by the same letter(s) are not significantly different according to Duncan’s multiple range test (*P* < 0.05)

### SEM studies of ZnO NPs and MgO NPs on the growth of *C. beticola*

**Fig. 3:**
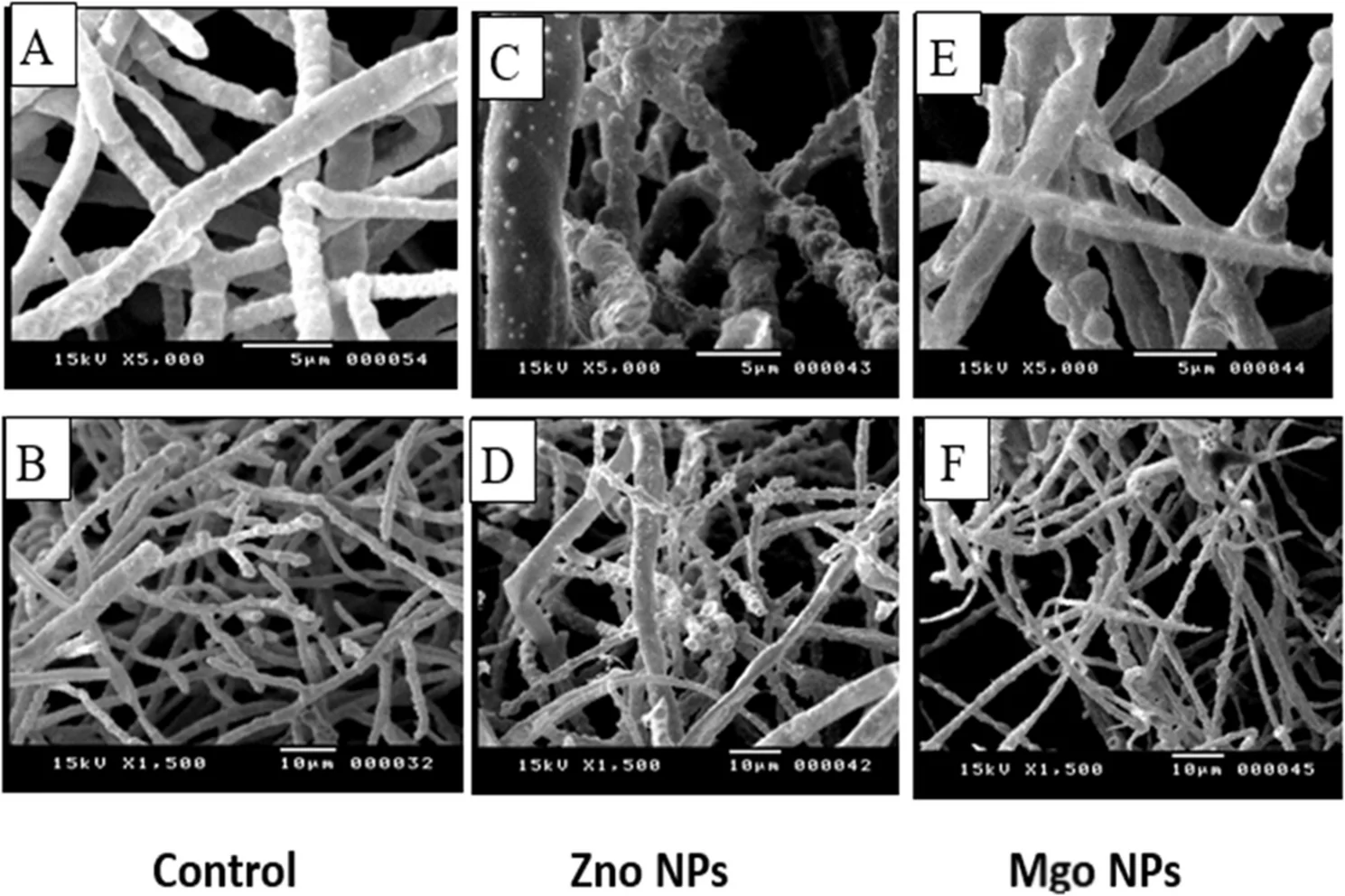
Scanning electron micrographs showing the ultrastructural effects of biosynthesized NPs on *C. beticola* hyphae at two magnification levels (×5000 and ×1500). **A**, **B** Untreated control *C. beticola* control. **C**, **D** Hyphae treated with ZnO NPs exhibiting distinct nodular surface formations and localized thickening. **E**, **F** Hyphae exposed to MgO NPs displaying mild surface roughness and slight structural irregularities

This study used SEM to observe the effects of chemically synthesized ZnO NPs and MgO NPs on the mycelial structure of *C. beticola.* Mycelia in the untreated control group were smooth, uniform and cylindrical, with complete, healthy structures (Fig. 3A&B). The surface of mycelia treated with ZnO NPs was covered by deposited nanoparticles, and the mycelia developed thickening and distortion. It is inferred that these NPs can directly act on and even penetrate fungal cell walls (Fig. 3C&D). Mycelia treated with MgO NPs exhibited swelling, nodular deformities and distortion. It is inferred that MgO NPs disrupt cell wall integrity to trigger physiological stress (Fig. 3E&F). Both types of nanoparticles damage the integrity of fungal cell walls and induced physiological stress.

### Effect of NPs on CLS disease of sugar beet under greenhouse conditions

**Fig. 4:**
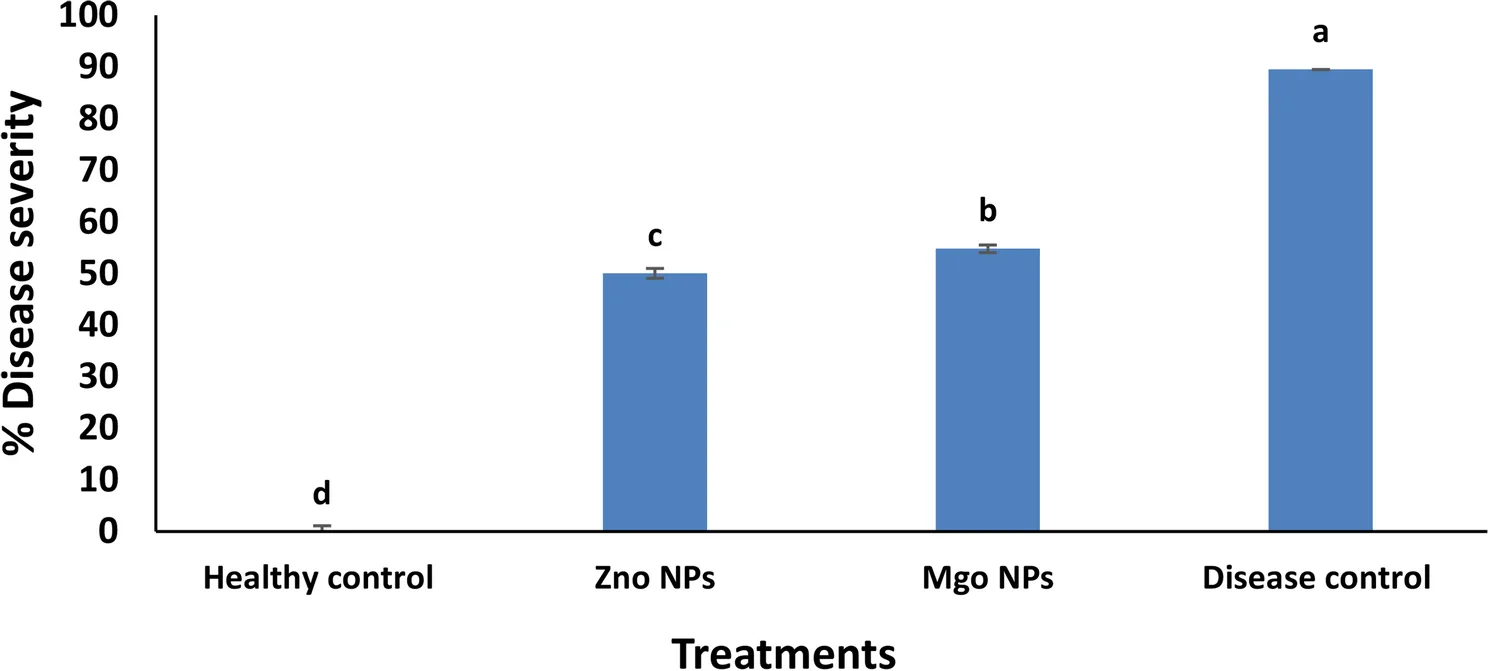
Effect of NPs on CLS under greenhouse conditions. Each point in the histogram represents mean value of three replicates and the vertical bars indicate + SE, which are significantly different at *P*< 0.05

This study conducted a greenhouse experiment (Fig. 4) to evaluate the efficacy of 90 ppm ZnO and MgO NPs in controlling sugar beet CLS disease. The disease control recorded the highest disease severity across all treatments, reaching 89.48%. Both treatment groups significantly reduced disease damage. The ZnO NPs delivered optimal performance with a disease severity of 50.00%, followed by the MgO NPs at 54.76%.

### Biochemical defense responses of sugar beet

#### Total phenolic compounds

**Fig. 5:**
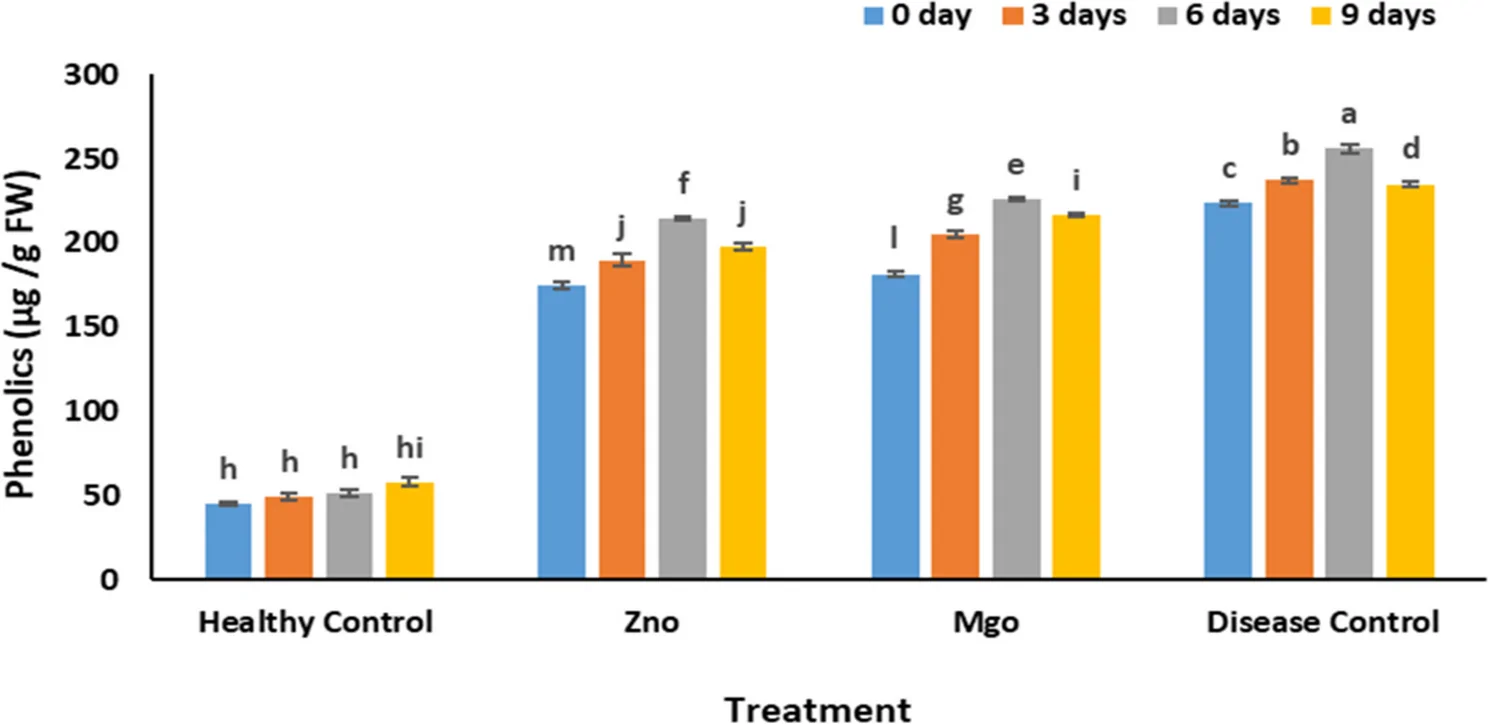
Effect of NPs on phenolic compounds of sugar beet plants at different time points (0 (day of treatment), 3, 6, and 9 days after treatment). Each point in the histogram represents mean value of three replicates and the vertical bars indicate + SE, which are significantly different at *P*< 0.05

Figure 5 presents the impact of ZnO NPs and MgO NPs on the total phenolic content in sugar beet leaves at four sampling times: 0, 3, 6, and 9 days under greenhouse conditions. The healthy control group recorded a total phenol content of only 51.09 mg/g, while the infected group inoculated with the sugar beet pathogen *C. beticola* reached 237.89 mg/g. This verifies that this pathogen can naturally trigger a strong defense response in plants. Treated with ZnO and MgO NPs, the phenol contents were 194.18 mg/g and 207.21 mg/g, respectively. Both treatments can effectively boost phenol accumulation, and their effect intensity is lower than the defense response induced by natural infection. Across the time dimension, the average phenol content was the lowest on day 0, at 156.25 µg/g, reached 170.28 µg/g on day 3, peaked at 187.06 µg/g on day 6, and dropped to 176.76 µg/g on day 9.

#### Peroxidase (PO) activity

**Fig. 6:**
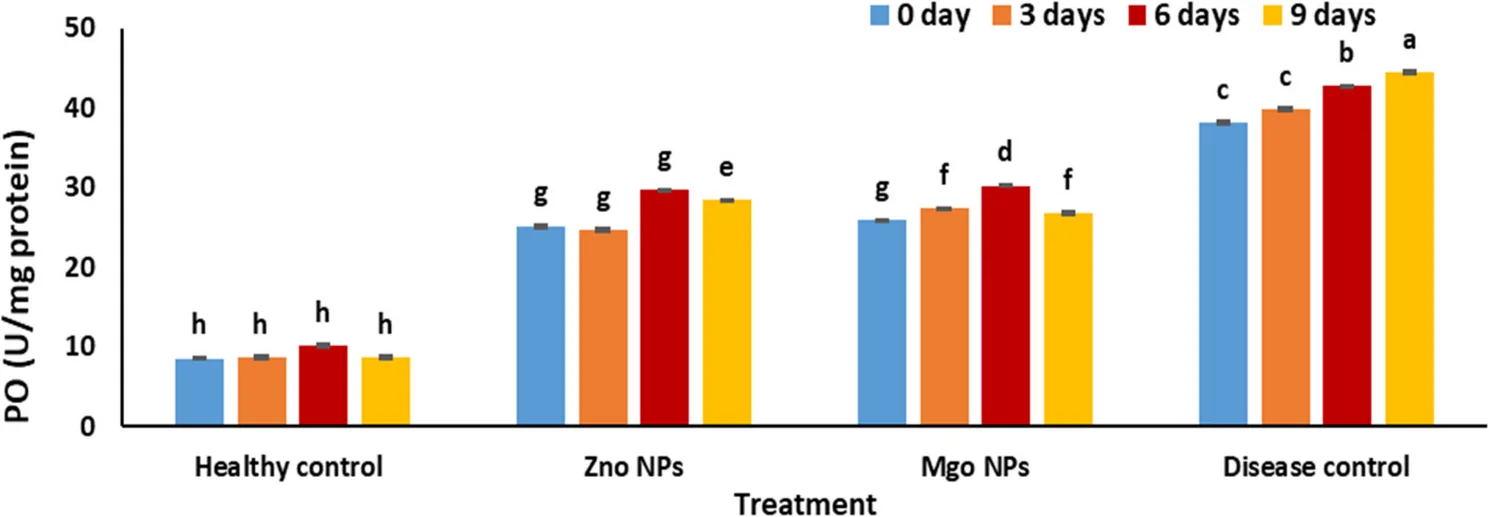
Effect of NPs on peroxidase (PO) of sugar beet plants at different time points (0 (day of treatment), 3, 6, and 9 days after treatment). Each point in the histogram represents mean value of three replicates and the vertical bars indicate + SE, which are significantly different at *P*< 0.05

The results in Fig. 6 clearly indicated that all treatments significantly increased peroxidase (PO) activity compared to the health control (9.02 U/mg protein), which recorded the lowest PO. In contrast, the disease control exhibited the highest PO activity (42.28 U/mg protein), reflecting the normal metabolic status of infected plants. The lowest level of PO was observed at the beginning of the experiment on 0 days (23.34 mg/g), followed by 3days (25.09 mg/g). Among the treatments, ZnO NPs exhibited the lowest PO activity in the infected plants (23.33 U/mg protein), followed by MgO NPs (28.63 U/mg protein). Regarding the impact of time, the highest PO activity was obsaeved after 6 days of treatment (28.81 U/mg protein), indicating that the metabolic activity in the treated plants reached its peak during this period. The PO levels showed a slight decrease by day nine (26.09 U/mg protein).

#### Polyphenol Oxidase (PPO) activity

**Fig. 7:**
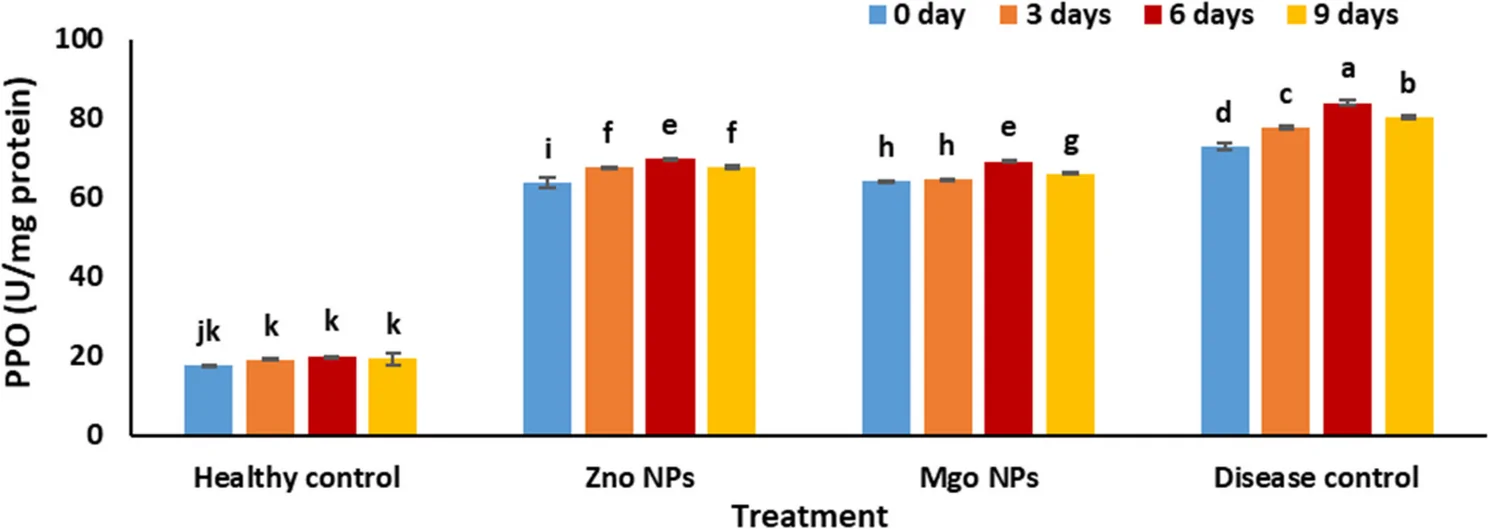
Effect of NPs on polyphenol oxidase (PPO) of sugar beet plants at different time points (0 (day of treatment), 3, 6, and 9 days after treatment). Each point in the histogram represents mean value of three replicates and the vertical bars indicate + SE, which are significantly different at *P*< 0.05

The treatments had differential effects on PPO activity (Fig. 7). All treated plants exhibited significantly higher PPO activity compared to the healthy control (19.02 U/mg protein). In contrast, ZnO NPs followed by MgO NPs produced higher PPO levels (67.15 and 65.95 U/mg protein, respectively), although these levels remained lower than those of the diseased control (80.17 U/mg protein).

With respect to time, PPO activity progressively increased after treatment, rising from 54.63 U/mg protein at 0 day to 56.22 after 3 days, then reaching a maximum at 6 days (60.70 U/mg protein), followed by a slight decline at 9 days (58.26 U/mg protein). This pattern reflects a typical induction followed by attenuation of the plant.

#### Phenylalanine Ammonia-lyase (PAL) activity

**Fig. 8:**
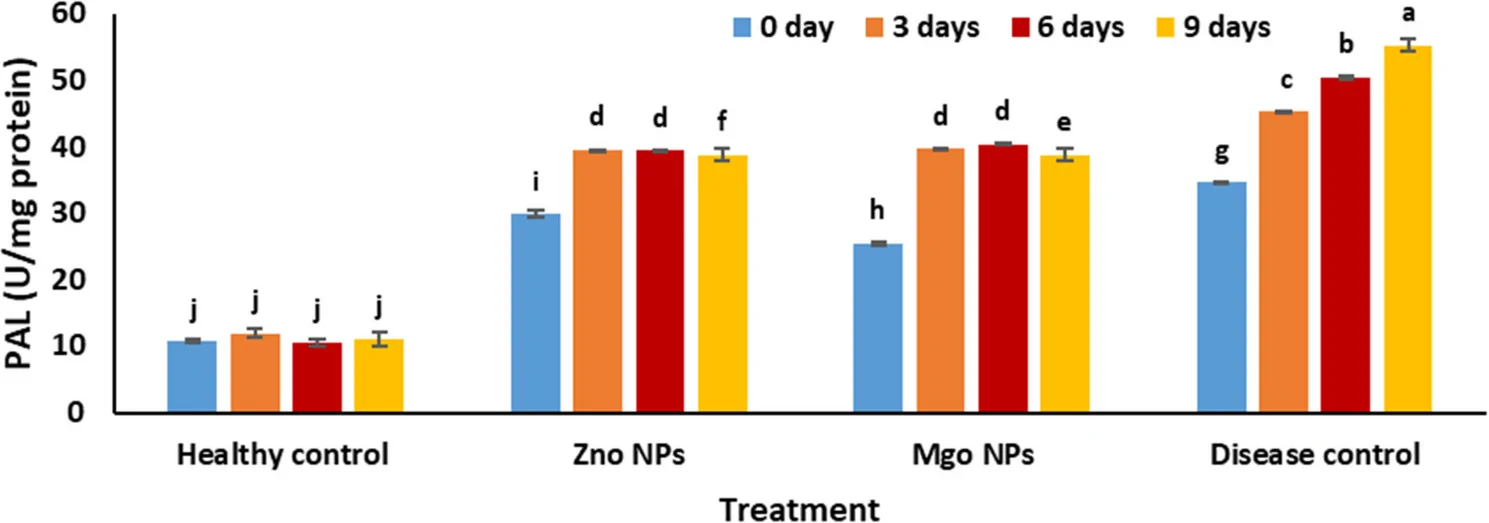
Effect of NPs on phenylalanine ammonia-lyase (PAL) of sugar beet plants at different time points (0 (day of treatment), 3, 6, and 9 days after treatment). Each point in the histogram represents the mean value of three replicates, and the vertical bars indicate + SE, which are significantly different at *P*< 0.05

The results demonstrated a clear improvement in PAL activity (Fig. 8) across all treated sugar beet compared to the healthy control (11.08 U/mg protein). Regarding the treatments used, ZnO NPs followed by MgO NPs showed the lowest PAL activity (36.35 and 33.85 U/mg protein, respectively) compared with the disease control (45.36 U/mg protein). With respect to the influence of time, the PAL activity increased at the beginning of the experiment at 0 day (25.13 U/mg protein). After three days, the level of PAL reached up to 32.81 U/mg protein. The highest level of PAL was recorded after 6 days of treatments (36.67 U/mg protein(. After 9 days, the PAL activity started to decrease (34.03 U/mg protein).

### Effect of NPs on CLS disease of sugar beet under field conditions

**Fig. 9:**
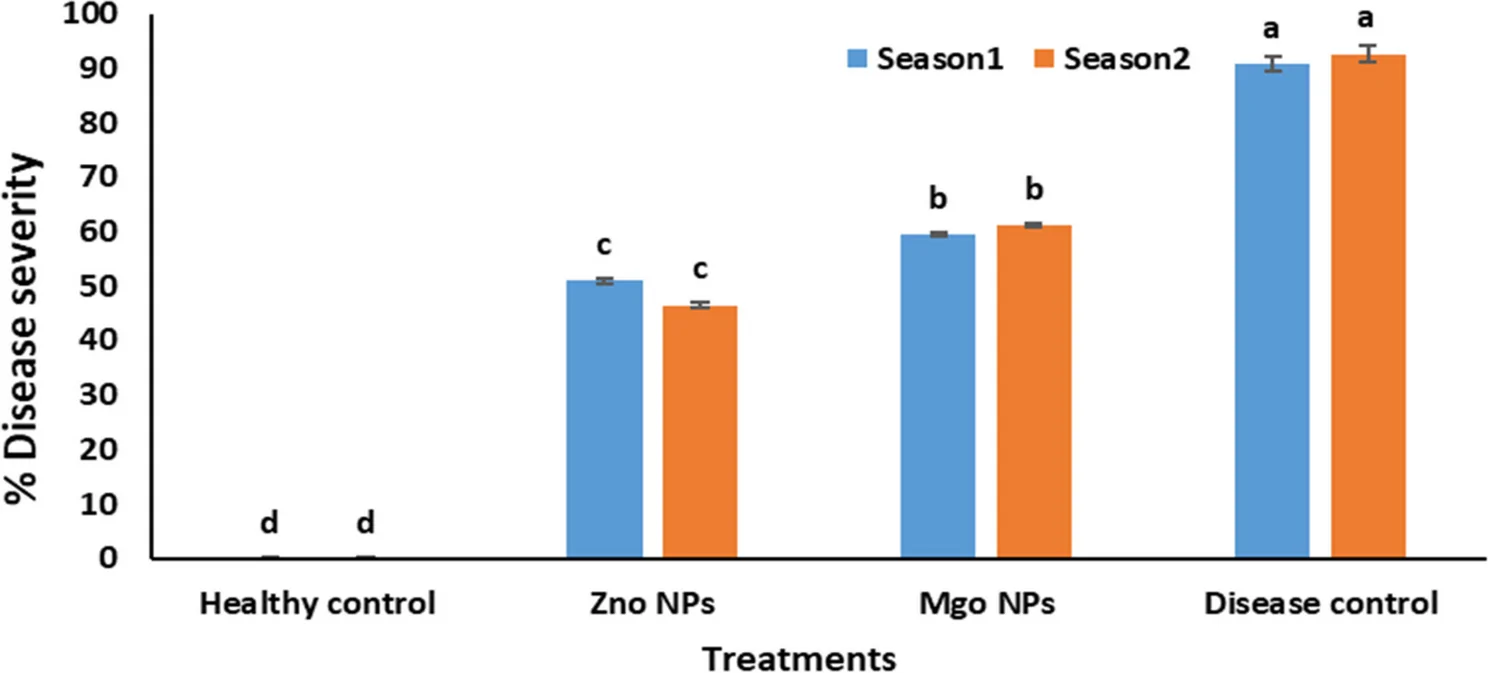
Effect of NPs on CLS disease under field conditions. Each point in the histogram represents mean value of three replicates and the vertical bars indicate + SE, which are significantly different at *P*< 0.05

The results clearly indicate (Fig. 9) that all treatments significantly decreased the severity of CLS disease compared to the diseased control, which recorded the highest values in both growing seasons (90.78% and 92.58%, respectively). In both growing seasons, treated sugar beet plants with ZnO NPs exhibited the highest disease suppression efficacy (50.92% and 46.52%, respectively). In comparison, MgO NPs were less effective at controlling disease severity (59.52% and 61.17%, respectively).

### Crop production under field conditions

During two growing seasons, the influence of NPs on sugar beet root yield was calculated under field conditions (Table 2). The results showed that all treatments significantly increased root yield compared to the diseased control (6.382 and 7.875 tons /feddan, respectively). The healthy control plants yielded amounts (9.495 and 11.182 tons/feddan, respectively). The highest root yield was recorded with ZnO NPs (10.118 and 11.707 tons/feddan, respectively), followed by MgO NPs (8.873 and 10.044 tons/feddan, respectively).

**Table 2:** Effect of NPs on crop production under field conditions

Root yield (tons/feddan)				
Treatments	Season1		Season2	
Healthy control	9.495	B	11.182	B
ZnO NPs	10.118	A	11.707	A
MgO NPs	8.873	C	10.044	C
Diseased control	6.382	D	7.875	D

Means defined in each column with the same letter(s) do not significantly according to the multiple range check by Duncan (*P* < 0.05). All values are the means of three replicates

### Analysis of sugar contents in sugar beet roots

The effect of ZnO NPs and MgO NPs on sugar accumulation in sugar beet roots was evaluated during two growing seasons (Table 3). Both reducing sugars and sucrose content were significantly affected by all treatments.

**Table 3:** Impact of NPs on sugar contents (reducing sugars and sucrose (%)) in sugar beet roots during two growing seasons (2023–2024 and 2024–2025)

Treatments	Season1				Season2			
	Reducing sugars (g/l)		Sucrose (%)		Reducing sugars (g/l)		Sucrose (%)	
Healthy control	0.83	a	19.27	A	0.517	a	19.77	a
Zno NPs	1.27	b	15.52	B	1.020	b	16.60	b
Mgo NPs	1.40	c	15.40	C	1.039	c	16.35	c
Diseased control	1.80	d	10.18	d	1.867	d	11.59 d	

Means defined in each column with the same letter(s) do not significantly differ according to the multiple range check by Duncan (*P* < 0.05). All values are the means of three replicates

In general, the diseased control exhibited the highest levels of reducing sugars (1.80 and 1.867 g/L, respectively) and the lowest sucrose content (10.18% and 11.59%, respectively). This reflects the negative effect of infection on sugar accumulation. Conversely, the healthy control recorded the lowest reducing sugar values (0.83 and 0.517 g/L, respectively) and the highest sucrose percentages (19.27% and 19.77%, respectively) in both seasons, representing the normal metabolic condition of uninfected plants.

ZnO NPs treatment resulted in higher reducing sugar contents (1.27 and 0.1020 g/L, respectively) and lower sucrose contents (15.52% and 16.60%, respectively). Similarly, MgO NPs treatment produced reducing sugar contents (1.40 and 1.039 g/L, respectively) and lower sucrose (15.40% and 16.35%, respectively), though still much better than the diseased control.

## Discussion

Sugar beet Cercospora leaf spot (CLS) is the core disease threatening global sugar beet production, causing extremely severe economic losses to the sugar beet industry. The current mainstream prevention and control model that relies on synthetic chemical fungicides has prominent environmental and health risks, creating an urgent need to develop new safe and sustainable prevention and control solutions. Nanoparticles (NPs) can boost antimicrobial activity due to the high surface energy associated with their nanoscale size. They have the dual functions of directly killing pathogens and activating plant defenses, and can effectively reduce the usage of chemical pesticides. Therefore, this study selected ZnO and MgO NPs as new prevention and control materials for CLS, and carried out experiments on their synthesis, characterization, and evaluation antifungal activity in vitro. This study synthesized the two types of nanoparticles to utilize their slow-release properties, and characterized their crystal structure, chemical composition, and micromorphology via XRD, FTIR, and TEM, respectively. XRD results demonstrated that the synthesized ZnO NPs are high-purity hexagonal crystals, which match the standard JCPDS card No. 01-080-0074, while the MgO NPs are cubic crystals matching JCPDS No. 01-075-0447. These crystal structure results are agree well with previous studies [13−23]. TEM observations show that ZnO NPs are a mix of semi-spherical and rod-shaped particles, while MgO NPs present an aggregated state, which also aligns with existing reports [24, 25]. It should be noted that the crystallite sizes estimated from XRD, and the particle dimensions observed by TEM represent different structural characteristics and therefore are not directly comparable. The crystallite size calculated from the Scherrer equation corresponds to the average size of coherently diffracting crystalline domains. Whereas TEM provides the overall particle morphology and physical dimensions. Accordingly, the ZnO nanorods observed by TEM, with an average length of approximately 958 nm, can consist of multiple smaller crystalline domains with an average crystallite size of 33.6 nm determined by XRD. Likewise, the aggregated morphology of MgO observed in the TEM images reflects the clustering of individual nanoparticles during synthesis or sample preparation rather than the size of single crystallites. Therefore, the apparent discrepancy between the XRD- and TEM-derived sizes is expected and is commonly reported for anisotropic and aggregated nanomaterials [26], where particle dimensions are significantly larger than the individual crystallite size.

In vitro antifungal tests were conducted in the current study at concentrations of 10, 30, 60, and 90 ppm. It was discovered that both types of NPs significantly inhibit *C. beticola* in a dose-dependent manner. The concentration with the greatest inhibitory effect was 90 ppm. The antifungal mechanism operates by using intense oxidation to generate reactive oxygen species (ROS) that damage the fungal cell membrane and impair metabolic functions [27, 28]. MgO NPs had a slightly better inhibitory effect than ZnO NPs, a difference that arises from the two materials’ differences in particle size, morphology, surface charge, and metal ion release capacity, which matches the conclusions of previous studies [29, 30].

SEM analysis was performed to examine the inhibitory effect of ZnO NPs and MgO on the target pathogenic NPs on *C. beticola*. In the control, fungal hyphae showed normal morphology and intact cell walls. Inthe ZnO NPs treatment, hyphae were severely deformed, with thickened cell walls and numerous nanoparticles deposited on their surfaces. These effects enhance localized oxidative stress and mechanical pressure, impairing membrane integrity and fungal viability [31, 32]. In the MgO NP treatment, hyphae became twisted, swollen, and tangled, accompanied by damaged conidia, suggesting inhibition of fungal reproduction through cell-wall destabilization [33, 34].

Greenhouse experiments were conducted to verify the efficacy NPs in controlling CLS in sugar beet. Disease severity across all treatments was significantly lower than in the untreated control. This study observed that ZnO and MgO nanoparticles can reduce plant diseases, which aligns with previously confirmed conclusions that metal oxide nanoparticles are effective against a wide range of plant fungal pathogens. Previous studies also documented that Nps inhibit the pathogenicity of pathogens: *Alternaria alternata* and *Colletotrichum gloeosporioides* [35]. This effect originates from both direct antifungal activity and the induction of host defense responses, which supports the application potential of integrating these materials into integrated disease management systems. Furthermore, the superior disease suppression observed in the present study can be explained by the intrinsic physicochemical characteristics of nanoparticles, particularly their high specific surface area, enhanced reactivity, and broad-spectrum antimicrobial properties, which facilitate greater interaction with fungal cells and improve biological efficacy [36, 37].

This study observed that NPs can activate the total phenolic content and the three defense enzymes, namely PO, PPO, and PAL, all increased, but their values were lower than those of the untreated infected control group. This study found that the elevated defensive enzyme activity detected in untreated infected plants arises from high-intensity pathogen stress, rather than the development of effective resistance. By contrast, plants treated with ZnO and MgO nanoparticles have their disease pressure reduced by the nanoparticles’ antifungal activity, so these plants do not need to over activate their own endogenous defense pathways [38]. Previous studies have suggested that NPs activate the phenylpropanoid pathway and the antioxidant defense system by inducing moderate ROS signaling, thereby enhancing defense enzyme expression. This type of response has been reported in previous studies on plant-pathogen interactions [37–39].

Existing studies have confirmed that the field control efficacy of ZnO NPs and MgO NPs against crop CLS disease is consistent with greenhouse experimental results. Compared with the infected control group, NPs treatment groups significantly reduced disease severity and increased crop root yield. These NPs boost yield increases by improving zinc availability, promoting chlorophyll synthesis, and regulating carbohydrate transport [40, 41]. Interestingly, MgO nanoparticles exhibited slightly greater antifungal activity than ZnO nanoparticles under in vitro conditions. However, in greenhouse and field application scenarios, ZnO NPs delivered far superior effects in disease suppression and crop growth promotion. Upon foliar application, ZnO can penetrate leaf tissues via its stable surface charge under field humidity conditions, and its ions can migrate efficiently to form systemic protection. By contrast, MgO is prone to aggregation, rendering it ineffective, and it is also vulnerable to being washed away and degraded [42].

The results further demonstrated that applying NPs can increase the sucrose content in sugar beet roots while reducing reducing-sugar content compared with the disease control. These effects may be associated with enhanced chlorophyll formation and photosynthetic activity, leading to higher sucrose production in leaves and greater transport to storage roots [43]. At the same time, they can enhance the activity of sucrose phosphate synthase (SPS) and sucrose synthase (SS), while suppressing invertase activity responsible for the breakdown of sucrose into reducing sugars (glucose and fructose). Accordingly, oxidative damage is minimized, enabling sugar metabolism to approach that of healthy plants, whereas carbohydrate metabolism is severely disrupted in infected plants [44, 45].

This study still has certain limitations that should be acknowledged. First, the disease prevention and control mechanisms of the ZnO and MgO NPs involved in this study are cited from earlier research, and three core items, including reactive oxygen species generation, have not been verified and need to be supplemented in follow-up work. Second, this study does not cover their long-term environmental impacts, and a comprehensive environmental risk assessment must be completed before large-scale application. Finally, standard chemical fungicides were not included in the reference treatment group. Therefore, we cannot directly conduct a side-by-side comparison between the antimicrobial efficacy of the NPs examined in this study and that of traditional fungicides. Future research should include this baseline fungicide reference to accurately assess the practical potential of these NPs in integrated disease management.

## Conclusion

This study confirms that ZnO NPs and MgO NPs can effectively control CLS disease. In vitro tests show that the antifungal activity of both NPs increases with rising concentration, and they can destroy the pathogen’s mycelium through physical mechanisms. In greenhouse and field trials, ZnO NPs deliver superior disease control performance, significantly reducing disease severity. Both types of NPs can increase the content of phenolic substances in sugar beets, and the activity of the core defensive enzymes PAL, PO, and PPO. Compared with the diseased control group, the treatment groups see significant increases in root yield and sucrose content, and a reduction in reducing sugar content. This study proposes that NPs are promising alternatives to conventional fungicides, and can be applied for the sustainable prevention and control of sugar beet leaf spot disease. However, relevant environmental assessments must be completed first before their large-scale agricultural application can be promoted. alternatives to conventional fungicides for the management of sugar beet Cercospora leaf spot.

## Data Availability

The datasets used or analyzed during the current study are available from the corresponding author upon reasonable request.

## Abbreviations

ZnO: Zinc Oxide
MgO: magnesium oxide
NPs: Nanoparticles
ZnO NPs: Znc Oxide Nanoparticles
MgO NPs: Magnesium Oxide Nanoparticles
CLS: Cercospora leaf spot
PAL: Phenylalanine Ammonia-lyase
PO: Peroxidase
PPO: Polyphenol Oxidase
C. beticola: Cercospora beticola
SEM: Scanning Electron Microscope
ROS: Reactive Oxygen Species
SPS: Sucrose Phosphate synthase
SS: Sucrose synthase
SBLEDA: Sugar beet leaf extract dextrose agar
XRD: X-ray diffraction
FTIR: Fourier transform infrared spectroscopy
TEM: Transmission electron microscopy
PDA: Potato dextrose agar
EMU: Electron Microscope Unit
DNS: Dinitro salicylic acid
%: Percent sign
°C: Celsius degrees
µg: Microgram
cm: Centimeter
cv: Cultivar
g: Gram
